# Epidemiology of dermatomycoses and onychomycoses in Ireland (2001–2020): A single‐institution review

**DOI:** 10.1111/myc.13473

**Published:** 2022-06-19

**Authors:** James Powell, Emma Porter, Sinead Field, Nuala H. O'Connell, Kieran Carty, Colum P. Dunne

**Affiliations:** ^1^ Department of Microbiology University Hospital Limerick Limerick Ireland; ^2^ School of Medicine and Centre for Interventions in Infection, Inflammation, and Immunity (4i) University of Limerick Limerick Ireland; ^3^ Department of Dermatology University Hospital Limerick Limerick Ireland

**Keywords:** dermatomycoses, dermatomycosis, epidemiology, Ireland, onychomycosis, tinea

## Abstract

**Background:**

Fungal skin infections are recognised as one of the most common health disorders globally, and dermatophyte infections of the skin, hair and nails are the most common fungal infections. Dermatophytes can be classified as anthropophilic, zoophilic or geophilic species based on their primary habitat association, and this classification makes epidemiological analysis useful for the prevention and control of these infections. The Irish contribution to the epidemiology of these infections has been scant, with just two papers (both reporting paediatric tinea capitis only) published in the last 20 years, and none in the last seven.

**Objectives:**

To perform a comprehensive retrospective epidemiological analysis of all dermatological mycology tests performed in University Hospital Limerick over a 20‐year period.

**Methods:**

All mycology laboratory test results were extracted from the Laboratory Information Management System (LIMS, iLab, DXC Technologies) from 2001 to 2020 inclusive for analysis. Specimen types were categorised according to the site of sampling. The data were analysed using Microsoft Excel.

**Results:**

About 12,951 specimens of skin, hair and nails were studied. Median patient age was 42 years (IQR 26–57) with a slight female preponderance (57.2%). Two thirds of samples (67%, *n* = 8633) were nail, 32% were skin scrapings (*n* = 4118) and 200 hair samples (1.5%) were received. Zoophilic dermatophytes were more commonly present in females (38% F, 23% M, proportion of dermatophytes) and in those under 10 years of age or from 45 to 70 years (36% and 34% zoophiles, respectively, proportion of dermatophytes), although anthropophiles predominated every age and gender category. Anthropophiles had their highest prevalence in the 10–20 years age category (80% anthropophiles, proportion of dermatophytes), and yeast infections were more prevalent in older patients (29% of >60 year olds vs. 17% of <60 year olds, proportion of all fungal positives). *Trichophyton rubrum* was the most prevalent pathogen detected, accounting for 53% of all dermatophytes detected, 61% of those detected from nail samples and 34% from skin and hair samples. *Trichophyton tonsurans* was the most prevalent dermatophyte in tinea capitis, accounting for 37% of dermatophytes detected. Both of these organisms are anthropophilic, and this group showed consistently increased prevalence in proportion to all fungal isolates. The proportion of this dermatophyte class (anthropophiles) increased among both nail samples and skin/hair samples during the study period, from 55% of samples in the first 5 years of the study (2001–2005) to 88% (proportion of dermatophytes) in the final 5 years. Conversely, yeast detection decreased.

**Conclusions:**

This study provides a detailed overview of the epidemiology of the fungal cultures of skin, nail and hair samples in the Mid‐West of Ireland over a 20‐year period. Monitoring this changing landscape is important in identifying likely sources of infections, to identifying potential outbreaks, and may help guide empiric treatment. To the best of our knowledge, this study provides the first detailed analysis from Ireland of fungal detections from skin, hair and nail samples, and is the first epidemiological fungal report of any kind in over 7 years.

## 1 INTRODUCTION

Fungal skin infections have been recognised as the fourth most common health disorder globally (after dental caries, tension‐type headaches and migraine),^1^ and dermatophyte infections of the skin, hair and nails are the most common fungal infections.^2^ Epidemiology of dermatophytes is relatively well understood. Specifically, dermatophytes can be classified according to their anthropophilic, zoophilic or geophilic nature based on their primary habitat association.^3^ Further, anthropophilic species have links to geographical regions,^2^ while zoophilic species have links to specific zoonotic hosts.^4^ Dermatophytoses caused by geophilic species are rare, and zoophilic species are often the most commonly detected. However, due to the changes in population mobility and lifestyle, there is an increasing shift toward predominance of anthropophilic species.^4^, ^5^, ^6^

Up to date knowledge of dermatophytes circulating locally and internationally can inform public health and veterinary interventions in reducing transmission of both anthropophilic and zoonotic dermatophyte infections.^7^ The emergence of anti‐fungal resistance, with an increasing number of reports of difficult to treat infections,^8^, ^9^, ^10^ has been highlighted as an issue of growing concern. Therefore, maintaining a high level of epidemiological oversight of these infections is crucial. Notably, the Irish contribution in this regard has been scant, with just two papers (both reporting paediatric tinea capitis only) published in the last 20 years,^6^, ^11^ and none in the last 7 years. Despite this limitation, there is an evident shift in this country from zoonotic to anthropophilic species associated with tinea capitis, increasing from just 51% anthropophilic in 1951^12^ (*n* = 70, Dublin and Cork) to 83.6% anthropophilic in 2006^6^ (*n* = 116, Dublin) and 97.8% anthropophilic in 2014^11^ (*n* = 192, Dublin). Many international studies mirror these findings, that is, anthropophilic species such as *Trichophyton tonsurans*, *Trichophyton violaceum* and *Trichophyton soudanense* have shown a propensity to be introduced by migrating populations and thrive in urban communities.^13^ Evidence of anthropophilic preponderance is evident in many parts of the world such as Spain,^14^ Portugal,^15^ Belgium,^16^ Poland,^17^ Iran,^18^ Mali,^19^ Senegal,^20^ Canada,^21^ Japan^22^ and Mauritania.^23^ In contrast, other regions remain predominated by zoophilic species, prinicipally *Microsporum canis* (e.g. Greece,^24^ Germany,^25^ Romania,^26^ Algeria,^27^ Argentina,^28^ Brazil,^29^ Korea^30^ and Taiwan^31^). It is also postulated that infections from some nominally zoophilic strains may indeed be of human origin, as evidenced by a clonal strain of *Trichophyton mentagrophytes* detected in a cluster of pubogenital infections in Germany for which no animal source has been found till date.^32^

In this context, the objective of our study was to analyse 20 years of epidemiological data regarding dermatological mycology testing performed in a large tertiary care teaching hospital in Ireland, and to determine whether the incidence of fungal infection and species involved have remained constant or altered significantly. This report represents the most comprehensive analysis of this type from Ireland, and it is hoped that its findings will be of interest to, and inform, researchers and clinicians focused on mycoses and international epidemiology.

## 2 METHODS

The authors confirm that the ethical policies of the journal, as noted on the journal's author guidelines page, have been adhered to and the appropriate ethical approval has been received from the Research Ethics Committee of University of Limerick Hospitals Group, Limerick, Ireland. All data accessed were anonymised, and individual patient consent was deemed not required.

The Department of Clinical Microbiology at University Hospital Limerick provides a centralised microbiology service for six acute hospital sites (867 current beds) and community healthcare facilities catering for a population of 473,269 people.^33^ Previous related research from our institution includes fungal bloodstream infections in our ICU patients, when an increased prevalence was noted when compared with our UK equivalents.^34^ For this study, all mycology laboratory test results from 2001 to 2020 were extracted from the Laboratory Information Management System (LIMS, iLab, DXC Technologies). For 16 ½ of those years testing was performed in‐house, for the remainder of the study period testing was outsourced due to staffing constraints in the laboratory. Due to the cost of outsourcing, some demand management measures were introduced in the laboratory which confined requests to Consultant Dermatologists and other practitioners with specialist training, and this reduced the number of tests performed during this latter period, this is discussed later as a limitation. Overall, the specimens originated mainly from General Practice (85%), but also from Dermatology clinics (9%), acute hospital in‐patients (1.6%) and out‐patients (2.7%). Specimen types were filtered for skin, hair and nail specimens only. The data were analysed using Microsoft Excel. Since the study was based solely on laboratory data we were unable to distinguish between relapses relative to specimens received from the same patient so the number of specimens was not corrected for duplicates. Specimens were tested by standard mycological procedures. Testing methodologies over the 20 year period varied, but in all cases where sufficient material was provided it consisted of direct microscopy for fungal elements using potassium hydroxide, and culture onto appropriate agar plates for 4 weeks. Identification of the isolated dermatophytes was based on the macroscopic and microscopic characteristics of the fungi. Yeast isolates were identified using API 20C (Biomerieux, France).

## 3 RESULTS

In the 20 year period studied, there were 12,951 specimens of skin, hair and nails examined for fungal analysis. The median age was 42 years (IQR 26–57 years) with a slight female preponderance (57.2%). A small number of samples had an unknown age (2 samples) and gender (24 samples). Two thirds (67%, *n* = 8633) were nail samples, of which 31% were from toes, 4% from fingers and 65% were unspecified. About 32% of samples were skin scrapings (*n* = 4118), of which 17% had no body site stated. 200 hair samples were received, of which 82% had an unspecified body site. The results from these specimens were categorised into dermatological investigations according to the following criteria:

- Onychomycosis: All nail samples
- Tinea capitis: All skin and hair samples from the head other than the face and all hair samples from unspecified body sites.
- Tinea corporis: All skin and hair samples other than those from hands, feet, head and groin.
- Tinea cruris: All skin and hair samples from the groin, inner thighs, penis, vulva and anus.
- Tinea facei/barbae: All skin and hair samples from the face.
- Tinea manuum: All skin and hair samples from the hands.
- Tinea pedis: All skin scrapings from the feet.
- Tinea (unspecified): All skin scrapings with no body site stated.

**FIGURE 1:**
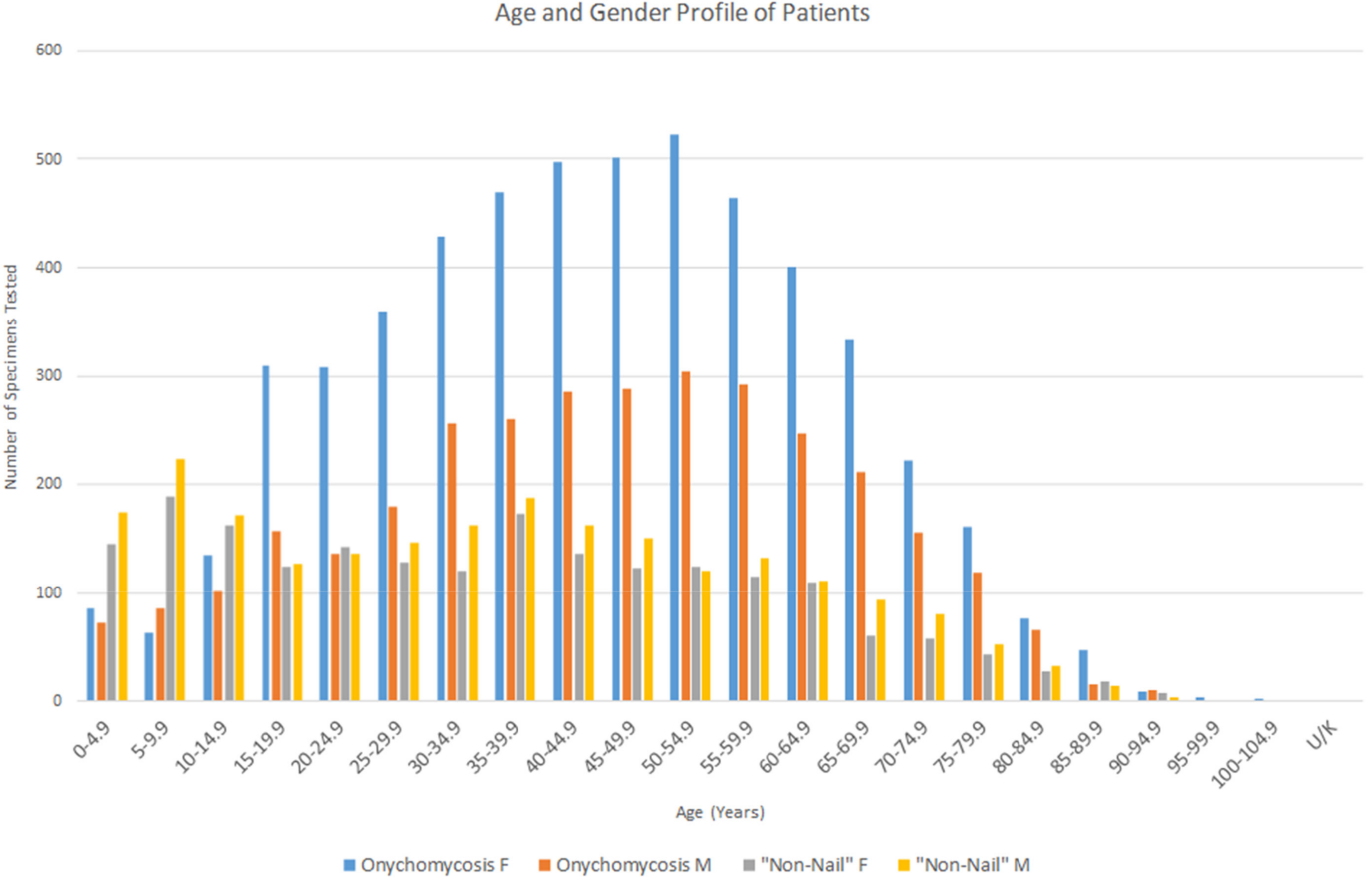
Distribution of patient age and gender for onychomycosis and all non‐nail tinea

Table 1 provides the age and gender breakdown according to each clinical diagnosis. Patients with fungal infection of the skin and hair collectively had a lower median age (34 years vs. 45 years), and a lower proportion of females (47% F vs. 63% F) than those with onychomycosis. The lowest age cohort was seen in the tinea capitis group, and the lowest female representation in the tinea cruris and tinea facei/barbae group. This age distribution was consistent over the period studied. Figure 1 depicts basic patient demographics for each category of fungal infection.

**TABLE 1:** Patient age and gender per site of infection. Median ages and % female were calculated using known values. A small number of patients were unknown

	Onychomycosis	T capitis	T corporis	T cruris	T facei/barbae	T manuum	T pedis
Age (years)							
*n*	8645	549	592	69	110	220	605
Median	45	10	40	40	32	39	36
IQR	31–59	5–30	22–58	26–58	10–45	29–53	18–53
%Female	62.5%	53.6%	42.2%	33.3%	40.0%	41.3%	46.2%

Nail specimens were more commonly collected from female patients (63% F vs. 37% M). Nail specimens from females had a lower yield of dermatophytes compared to specimens from males (15% F vs. 25% M), but a slightly higher proportion of yeasts (6% F vs. 5% M). Skin and hair samples were more commonly collected from male patients (53%), although gender distribution for fungal positivity was equal (19%) on testing. Of the fungal positive skin and hair samples, the proportion of those that were dermatophytes is also equal (79%) across genders. Among all specimens, zoophilic dermatophytes were more commonly present in females (38% F, 23% M, proportion of dermatophytes). This preponderance of zoophiles among the detected dermatophytes was most evident among the tinea capitis (22 of 42 F patients vs. 18 of 58 M patients) and tinea corporis (13 of 24 F patients vs. 10 of 40 M patients) groups.

Table 2 provides mycology results categorised by age and infection type. For convenience, the onychomycosis group is compared with all non‐nail sites of fungal infections collectively. Anthropophiles were more prevalent in the younger cohort of the onychomycosis group (83% of dermatophytes in those under 30 years vs. 69% in those over 30 years of age), but the reverse is the case in the non‐nail group (54% in the under 30 s vs. 65% in the over 30 s).

**TABLE 2:** Infectious species collated by age and infection type (broad)

	Onychomycosis (age in years)					Tinea–All Body Sites (age in years)				
	0–14	15–29	30–64	≥65	Total	0–14	15–29	30–64	≥65	Total
Anthrophiles										
*Epidermophyton floccosum*	0	1	8	5	15	8	1	3	0	12
*Microsporum audouinii*	0	1	0	0	1	2	0	1	0	3
*Microsporum ferrugineum*	0	0	1	0	1	0	0	0	1	1
*Trichophyton interdigitale*	5	26	82	17	146	3	7	31	7	48
*Trichophyton rubrum*	87	234	485	98	984	18	50	114	38	220
*Trichophyton schoenleinii*	0	0	0	0	0	3	0	0	0	3
*Trichophyton soudanense*	0	0	0	1	1	1	0	0	0	1
*Trichophyton tonsurans*	1	3	14	4	23	69	8	7	3	87
*Trichophyton violaceum*	1	1	0	0	2	10	0	0	0	10
Zoophiles										
*Microsporum canis*	1	0	3	0	4	75	10	18	7	110
*Trichophyton mentagrophytes*	5	59	241	66	421	13	26	68	7	114
*Microsporum persicolor*	0	0	1	0	1	2	0	0	0	2
*Trichophyton verrucosum*	0	4	2	0	9	13	6	5	2	26
Other										
*Microsporum gypseum*	0	0	0	0	0	2	1	0	0	3
Trichophyton species (No ID)	4	1	4	0	9	3	0	2	1	6
Yeast	27	76	273	118	494	16	26	61	21	124
Non‐Derm Mould	4	17	124	58	203	2	6	23	5	36
Mixed Dermatophytes	0	0	1	0	1	1	0	0	0	1
Mixed non‐dermatophytes	0	1	6	9	16	0	0	3	2	5
Microscopy Pos, Culture Neg	63	165	629	190	1047	29	33	90	13	165
Negative	330	848	3157	846	5182	757	611	1438	379	3187
Insufficient/Contaminated	15	14	39	17	85	45	27	64	6	142
Total	543	1451	5221	1429	8645	1072	812	1928	492	4306

The median age of patients with zoophilic dermatophytes from skin and hair samples was 24.5 years (IQR 8–44 years), which was younger than that seen from the anthropophilic dermatophytoses of skin and hair (median 33 years, IQR 11–51). The reverse was identified from those with onychomycoses, where the median age of patients with zoophilic dermatophytes (49 years, IQR 35–59 years) was older than those with anthropophilic dermatophytes (median 39 years, IQR 26–53 years).

**FIGURE 2:**
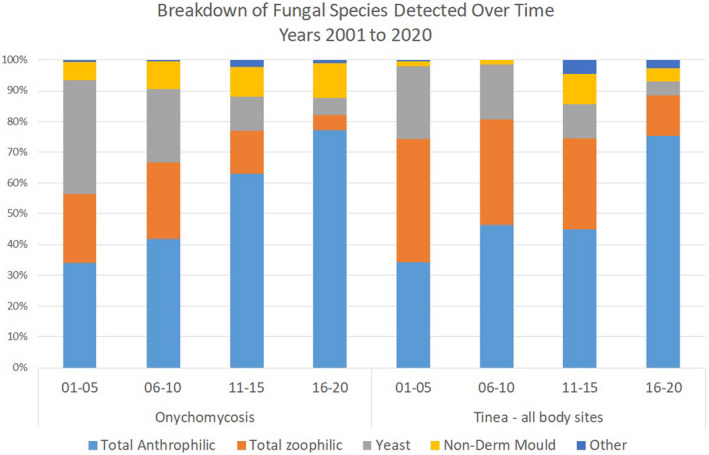
Distribution (proportions) of fungal categories over time, broken into four equal 5‐year periods from 2001 to 2020

A decline in the detection of zoophiles over each of the four five‐year periods of the study (44%, 39%, 22%, 10%, proportion of dermatophytes, respectively), and the inverse for anthropophiles (55%, 61%, 76%, 88%) was noted. This trend occurred across both the onychomycosis and non‐nail groups. Yeast detections were also consistently lower across each of the four quarters of the 20 year study, in both the onychomycosis (37%, 24%, 11%, 5%, proportion of all fungal positives) and the non‐nail group (24%, 18%, 11%, 4%). See Figure 2 for a chart demonstrating these trends. The remainder of the fungal detections comprise non‐dermatophyte moulds, mixed cultures and cultures without a definitive identification.

Yeast infections were more prevalent in the older populations of both groups, detected in 31% of nail and 22% of non‐nail fungal positive specimens in those over 65, and 19% and 14% specimens, respectively, in those under 65.

Table 3 shows a more complete breakdown of test results by category of infection. *Trichophyton rubrum* was the most prevalent pathogen detected, accounting for 60% of dermatophytes detected in onychomycosis and tinea pedis, 83% of tinea cruris, 68% of tinea manuum, 44% of tinea corporis, 10% of tinea faciei/barbae and 9% of tinea capitis. *Trichophyton interdigitale* was the next most commonly detected anthropophilic dermatophyte, accounting for 9% of all dermatophytes, and 19% of those detected from tinea pedis. *Trichophyton tonsurans* was the most prevalent dermatophyte in tinea capitis, accounting for 37% of dermatophytes detected from these specimens and 5% of all dermatophytes. These three anthropophilic organisms represented 67% of all dermatophytes detected.

**TABLE 3:** Infection type (detailed) correlated with identified species

	Onychomycosis	Tinea capitis	Tinea corporis	Tinea cruris	Tinea facei/barbae	Tinea manuum	Tinea pedis	s(Unspecified)	Total
Anthrophiles									
*Epidermophyton floccosum*	15	2	1	1	0	1	1	6	27
*Microsporum audouinii*	1	2	0	0	1	0	0	0	4
*Microsporum ferrugineum*	1	0	1	0	0	0	0	0	2
*Trichophyton interdigitale*	146	0	2	0	1	1	25	19	194
*Trichophyton rubrum*	984	9	28	5	1	13	58	106	1204
*Trichophyton schoenleinii*	0	3	0	0	0	0	0	0	3
*Trichophyton soudanense*	1	0	0	0	0	0	0	1	2
*Trichophyton tonsurans*	23	37	6	0	3	1	1	39	110
*Trichophyton violaceum*	2	6	0	0	0	0	0	4	12
Zoophiles									
*Microsporum canis*	4	35	14	0	1	0	0	60	114
*Trichophyton mentagrophytes*	421	2	5	0	0	1	49	57	535
*Microsporum persicolor*	1	0	0	0	0	0	0	2	3
*Trichophyton verrucosum*	9	3	4	0	2	2	0	15	35
Other									
*Microsporum gypseum*	0	0	1	0	0	0	0	2	3
*Trichophyton species (No ID)*	9	1	2	0	1	0	0	2	15
Yeast	494	5	18	4	2	6	20	69	618
Non‐Derm Mould	203	2	6	0	3	1	6	18	239
Mixed Dermatophytes	1	0	0	0	0	0	0	1	2
Mixed non‐dermatophytes	16	0	3	0	0	0	0	2	21
Microscopy Pos, Culture Neg	1047	8	19	3	8	4	25	98	1212
Negative	5182	428	459	55	85	179	413	1568	8369
Insufficient/Contaminated	85	6	23	1	2	11	7	92	227
Total	8645	549	592	69	110	220	605	2161	12,951

*Trichophyton mentagrophytes* was the most prevalent zoophilic dermatophyte, representing 24% of all dermatophyte detections. The majority of the *T. mentagrophytes* detections (88%) were from onychomycosis and tinea pedis patients, where this organism accounted for 27% of dermatophytes. For the remainder of the specimens (skin and hair specimens other than from tinea pedis), *T. mentagrophytes* accounted for 13% of dermatophyte detections. *Microsporum canis* was the next most commonly detected zoophilic dermatophyte, representing 5% of all dermatophytes detected. The majority of those detections were from tinea capitis and tinea corporis (65% and 26%, respectively, of *M. canis* detections from specimens with a body site stated), where *M. canis* was the second most commonly detected dermatophyte in both categories.

## 4 DISCUSSION

To the best of our knowledge, we provide the first detailed analysis from Ireland of fungal detections from all external body sites (skin, hair and nail) from this country in over 30 years, and the first from Britain and Ireland in more than 10 years. There are no previous published epidemiological reports of onychomycosis detections from Ireland. The most recent paper from this country on dermatophytoses^11^ reports an increasing shift from zoophilic species to anthropophilic species, and our data support this observation. The most recent reports from all body sites from other centres in Ireland are one from Dublin and Cork in 1951,^12^ and one from Cork in 1981^35^; they describe zoophilic species accounting for 48% and 59% of dermatophyte infections, respectively. Our analysis found 39% zoophiles overall among fungal positive skin and hair specimens, but this reduced from 54% (95 of 177 specimens) in the first 5 years (2001–2005) to just 15% in the last 5 years (19 of 129 specimens in 2015–2020).

More contemporary reports of tinea capitis specifically originated from Dublin in 2006^6^ and 2014,^11^ when the proportion of zoophiles among dermatophytes were 15% and 2%, respectively. Our comparable level among tinea capitis specimens was 40% (40 of 100 specimens), albeit that this proportion reduced over time from 58% (18 of 31) of tinea capitis dermatophytes being zoophiles in the first 5 years of the study to just 36% (8 of 22) in the last 5 years. Our region of Ireland (the Mid‐West) comprises a more rural population (54% rural) than Dublin City (2% rural).^36^ The Mid‐West of Ireland is also more ethnically indigenous and homogenous, with 97% of respondents of the 2016 census describing themselves as having a EU Nationality compared with 90% of respondents from Dublin City (89% and 80% respectively described as “Irish”).^33^, ^37^ These demographic differences may account for a higher rate of zoophiles (and a lower proportion of dermatophytes associated with migrating populations) seen in our patients than those in the studies from the country's capital.

Much of the Mid‐West of Ireland is rural and agricultural, with a bovine population^38^ outnumbering that of humans^33^ by more than 2:1. Uptake of the licenced vaccine for cattle ringworm (Bovilis® Ringvac) is low, and anecdotal evidence of visible ringworm among livestock is commonplace. Given this scenario, it is noteworthy that *Trichophyton verrucosum*, the dermatophyte with the highest prevalence in ruminant animals,^39^ was detected infrequently among our dermatophytes (1.5%, 35 of 2265), and was detected just once in the last 5 years of the study.

International reports on tinea capitis state that *Microsporum canis* and *Trichophyton tonsurans* are the two main pathogens in Europe, the former predominating in Mediterranean countries and the latter in the UK, our nearest neighbour.^38^ Our data are accordant with these findings; these two organisms account for 72% of our tinea capitis organisms (35 and 37 detections from 100, respectively). The African strains *Trichophyton soudanense* and *Trichophyton violaceum* have been identified in a small number of specimens, mainly paediatric (12 of 14 positive specimens were from paediatric patients) and tinea capitis patients (6 of 9 specimens with a site stated were from the scalp, five detections were from skin scrapings with no site stated, two were from nail clippings), accounting for 0.6% of the dermatophytes detected. Only 0.8% of the 2016 population in this region described themselves as ‘Black or Black Irish’.^33^

The prevalence of dermatophytes (69%, 1618 of 2331 fungal isolates) in our onychomycosis specimens was consistent with other studies in temperate climates, in contrast to tropical and subtropical climates where the leading aetiological agents are known to be non‐dermatophytes and yeast.^39^, ^40^ The median age of the total onychomycosis patients (45 years) and the median age of those with positive fungal cultures (42 years) are lower than that seen in other studies.^39^, ^41^ The preponderance of females among the onychomycosis patients (63% of patients were female) has also been a feature of previous studies, but studies showing the reverse and others with an equal proportion have also been reported.^42^

A limitation of this study is that only classical methods were used for the identification of the isolated fungi. Another limitation is that the body site was not clarified for 17% of skin and hair samples, and 65% of nail samples. Finally, the availability of mycology testing was not consistent throughout the study period, with curtailment and outsourcing of services applied in July 2016 due to staff shortages.

Irrespective, the strength of this study is provision of a detailed overview of the epidemiology of the fungal cultures of skin, nail and hair samples in the Mid‐West of Ireland over a 20‐year period. Few studies of this scale are available from Britain and Ireland, save the compendious analysis of Borman et al^43^ in 2007, with none in the last decade. A search of the literature for the last 5 years identifies over 50 original articles giving an epidemiological analysis of dermatophyte detections worldwide. Many cover ss alone,^15^, ^18^, ^19^, ^21^, ^23^, ^24^, ^27^, ^29^, ^30^, ^31^, ^44^, ^45^, ^46^, ^47^, ^48^, ^49^, ^50^ others cover onychomycosis and tinea pedis alone,^39^, ^40^, ^41^, ^50^, ^51^, ^52^, ^53^, ^54^, ^55^, ^56^, ^57^, ^58^, ^59^, ^60^, ^61^, ^62^, ^63^ including the only article in this search from the UK and Ireland.^61^ Eighteen articles were identified where all dermatomycoses were analysed,^14^, ^16^, ^17^, ^20^, ^22^, ^26^, ^28^, ^64^, ^65^, ^66^, ^67^, ^68^, ^69^, ^70^, ^71^, ^72^, ^73^, ^74^ but just five of these were of a 10 year duration or longer^14^, ^65^, ^66^, ^71^, ^72^ where trends could be identified. Fungal epidemiology is dynamic internationally, and this study further demonstrates the changing prevalence of different mycoses over time. This information is important for the identification of the likely source of fungal infections and outbreaks, and in guiding empiric treatment.

## AUTHOR CONTRIBUTIONS

JP involved in writing—original draft (lead); data curation (lead); methodology (lead) and writing—review and editing (equal). EP performed writing—original draft (supporting) and writing—review and editing (equal). SF involved in writing—original draft (supporting) and writing—review and editing (equal). NOC involved in conceptualisation (lead); writing—original draft (supporting) and writing—review and editing (equal). KC involved in conceptualisation (supporting) and review and editing (equal). CPD involved in conceptualisation (supporting); writing—original draft (supporting) and review and editing (equal).

## CONFLICT OF INTEREST

The authors certify that they have no affiliations with or involvement in any organisation or entity with any financial interest, or non‐financial interest in the subject matter or materials discussed in this manuscript.
